# *Sucnr1* Mediates Leukocyte–Endothelial Cell Interactions Induced by Tnfα

**DOI:** 10.3390/ijms27177930

**Published:** 2026-09-05

**Authors:** Sandra Coll, Ana Jarén, Cristina Bauset, Dulce C. Macias-Ceja, Dolores Ortiz-Masiá, Jesús Cosín-Roger, María D. Barrachina, Sara Calatayud

**Affiliations:** 1 Departamento de Farmacología, Facultad de Medicina, Universidad de Valencia, 46010 Valencia, Spain; sandra.coll@uv.es (S.C.); ana.m.crespo@uv.es (A.J.); cristina.bauset@uv.es (C.B.); dulce.macias@uv.es (D.C.M.-C.); jesus.cosin@uv.es (J.C.-R.); sara.calatayud@uv.es (S.C.); 2 Departamento de Medicina, Facultad de Medicina, Universidad de Valencia, 46010 Valencia, Spain; m.dolores.ortiz@uv.es; 3 Biomedical Research Center for Liver and Digestive Diseases (CIBEREHD), 46010 Valencia, Spain

**Keywords:** succinate, SUCNR1, TNFα, leukocyte–endothelial interactions, inflammation

## Abstract

Succinate is a metabolite involved in chronic inflammatory diseases, regulating macrophages, dendritic cells, and lymphocytes via its receptor SUCNR1 and through intracellular pathways. Our aim was to analyze whether the succinate–SUCNR1 axis modulates the leukocyte–endothelium interactions (L/EI) that mediate the formation of inflammatory foci in response to a ubiquitous pro-inflammatory cytokine (TNFα). L/EI were analyzed in murine cremasteric venules *in vivo* and between human umbilical endothelial cells (HUVECs) and peripheral blood mononuclear cells (PBMCs) *in vitro*. We observed that TNFα increased the expression of SUCNR1 and that the L/EI, the proinflammatory cytokines’ upregulation and the NF-κB activation that induced this cytokine were reduced in *Sucnr1*^−/−^ mice in comparison with WT mice. Intrascrotal injection of exogenous succinate did not induce significant proinflammatory effects *per se* but, combined with TNFα, allowed a *Sucnr1*-independent upregulation of pro-inflammatory cytokines. In vitro, the SUCNR1 antagonist NF-56-EJ40 prevented the interactions of PBMCs with TNFα-treated HUVECs. HUVECs incubated with exogenous succinate presented higher L/EI but a limited response to TNFα. SUCNR1 contributes to TNFα-induced leukocyte–endothelial interactions. However, exogenous administration of high concentrations of this succinate exerts a complex pattern of effects that may include anti-inflammatory actions.

## 1. Introduction

Evidence accumulated over the last decades establishes an important bond between metabolism and the inflammatory and immune responses, giving rise to the concept of immunometabolism [1]. Changes in the activity of immune cells require, and are associated with, metabolic adaptations. In parallel, the fluctuations in metabolic intermediate levels may influence the activity of immune cells [2] by acting on the growing group of metabolite-sensing receptors [3,4]. One of these signaling metabolites is succinate [5,6]. This Krebs cycle intermediate is generated from succinyl-CoA, which in turn can arise from glutaminolysis, the GABA shunt, and the catabolism of specific amino acids. Succinate is subsequently oxidized to fumarate by succinate dehydrogenase (SDH), which also functions as complex II of the mitochondrial electron transport chain. Thus, SDH provides a direct link between the Krebs cycle and the respiratory chain, making succinate metabolism responsive to multiple regulatory factors [6,7]. In fact, succinate levels are increased in varied physiological and pathological conditions [8,9,10], including common inflammatory disorders like rheumatoid arthritis [11], inflammatory bowel disease [12] or obesity [13]. Several biochemical outputs with proinflammatory potential have been described as consequences of succinate accumulation [14]. Within the mitochondria, elevated succinate drives electron flow through complex II, leading to ubiquinone reduction and reverse electron transport (RET) through complex I to generate reactive oxygen species (ROS). In the cytosol, elevated succinate exerts product inhibition on α-ketoglutarate-dependent dioxygenases (α-KGDDs), including prolyl hydroxylase domain enzymes that target HIF-1α for degradation. This inhibition promotes HIF-1α stabilization and drives downstream inflammatory responses, including interleukin-1β (IL-1β) production [15,16]. Finally, succinate can exit to the extracellular milieu where it can function as a signaling molecule by stimulating the G-protein coupled receptor SUCNR1 [17]. Contrasting with these phlogogenic activities, anti-inflammatory properties have been assigned to succinate in specific contexts [14,18,19,20].

The role of this signaling pathway on inflammation has been essentially attributed to the modulation of immune cells like macrophages, dendritic cells and lymphocytes [14]. However, the range of cells that may be regulated by succinate goes beyond leukocytes and includes, amongst others, the endothelial cells [21,22,23]. In fact, previous reports demonstrate that this metabolite acts on the vascular endothelium to modulate the process of angiogenesis [24,25] or to regulate vascular tone [26]. The present study analyses whether succinate and the activation of its receptor affect the key role that endothelial cells play in mediating the homing and accumulation of leukocytes, and so, allowing the formation of inflammatory foci. To this end, we have studied the mouse cremasteric tissue by intravital microscopy, a widely used model for investigating the microcirculation *in vivo* [27], as well as the interactions between human endothelial cells and leukocytes *in vitro* under dynamic conditions.

## 2. Results

### 2.1. Role of the Succinate-SUCNR1 Signaling Axis on the Formation of an Inflammatory Response Induced by TNFα In Vivo

**Figure 1:**
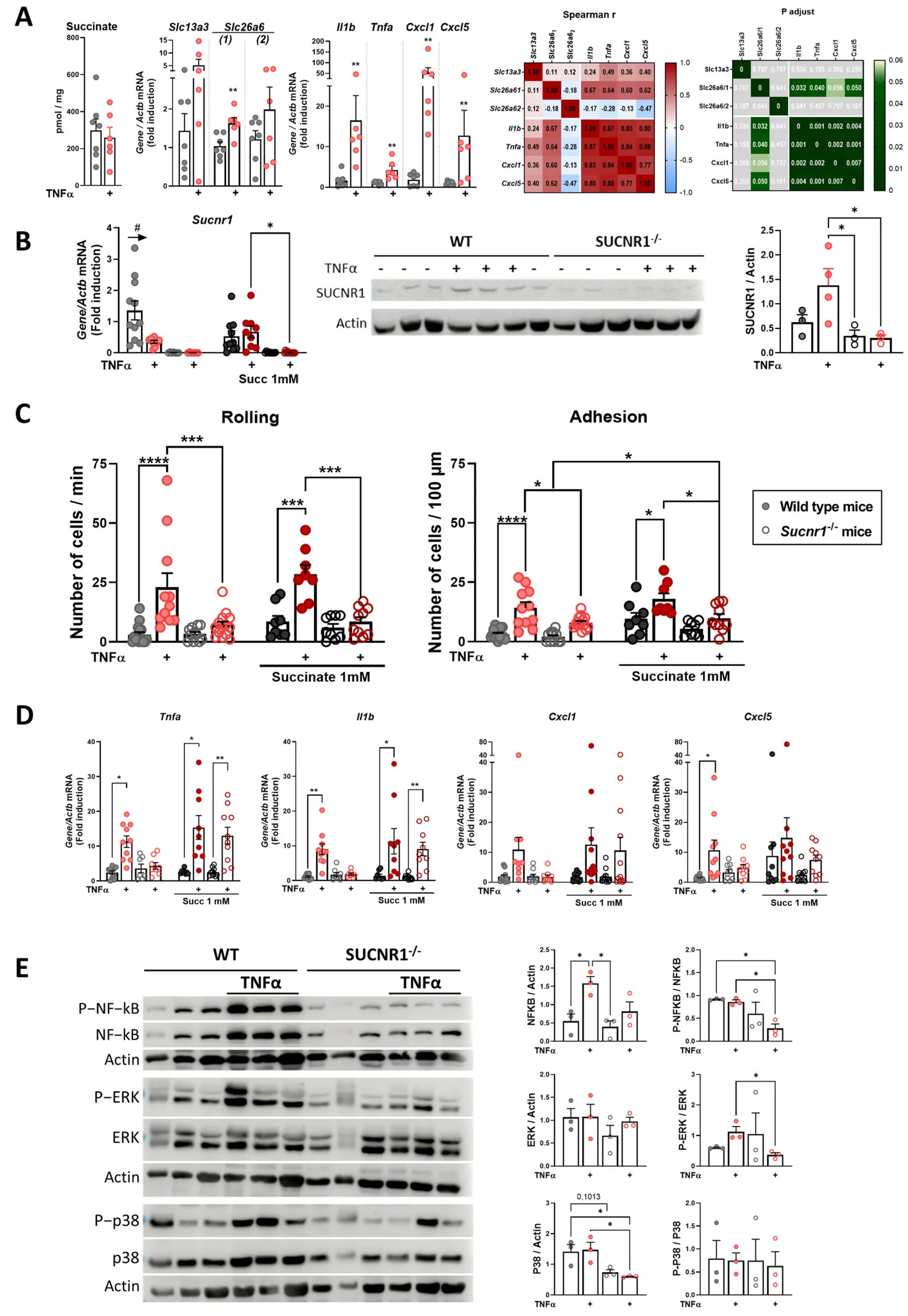
Effects of TNFα in wild-type and *Sucnr1* knockout mice. Animals were treated with TNFα (500 ng, i.p.), with succinate (1 mM, intraescrotally) or their vehicles, and their cremasteric tissues were analyzed 4 h later for: (**A**) succinate levels and gene expression of the anion transporters *Slc13a3* and *Slc26a6* (isoforms 1 and 2) and of cytokines/chemokines (the heatmaps show a correlational study of the transcriptional data, Spearman’s R and *p*-adjust values) (n = 6–7); (**B**) gene (n = 8–15) and protein (n = 3–4) expression of SUCNR1; (**C**) leukocyte rolling flux and adhesion in cremasteric venules (intravital microscopy, number of cells per 100 μm of vessel) (n = 8–15); (**D**) gene expression of cytokines/chemokines (n = 8–15); and (**E**) signaling protein levels (n = 3). Results are mean ± SEM., * *p* < 0.05, ** *p* < 0.01, *** *p* < 0.001 and **** *p* < 0.0001 vs. indicated group, # *p* < 0.05 vs. all the other groups ((**A**): Student’s T-test; (**C**,**E**): ANOVA followed by Tukey test: (**D**): Kruskal–Wallis followed by Dunn’s test).

The administration of TNFα (500 ng, i.p.) did not modify succinate levels in the cremasteric tissue. However, this cytokine increased the gene expression of *Slc26a6*, a solute carrier that favors the presence of this carboxylic acid in the extracellular milieu [16,28,29], and whose expression showed positive and significant correlations with that of pro-inflammatory cytokines and chemokines (Figure 1A).

The administration of TNFα was associated with a significant increase in SUCNR1 protein levels in the cremasteric tissue. This protein was not detected in *Sucnr1*^−/−^ mice. Analysis of *Sucnr1* mRNA expression confirmed its absence in *Sucnr1*^−/−^ mice and detected a downregulation of *Sucnr1* gene expression in wild-type mice treated with TNFα (Figure 1B).

#### 2.1.1. Leukocyte–Endothelial Cell Interactions

The intravital experiments showed that the increase in leukocyte rolling and adhesion induced by TNFα was significantly reduced in *Sucnr1*^−/−^ mice (Figure 1C, Videos S1 and S2). The intraescrotal injection of succinate (1 mM) was associated with a non-significant increase in leukocyte–endothelial interactions in wild-type mice. The response elicited by the combination of TNFα plus succinate was significantly reduced by the absence of SUCNR1, although in the case of leukocyte adhesion the reduction was only partial (Figure 1C), suggesting that this effect is mediated by both SUCNR1-dependent and -independent pathways.

#### 2.1.2. Cytokine Expression

The gene expression of pro-inflammatory cytokines and chemokines in the scrotal tissue of wild-type mice was increased by TNFα but unaffected by succinate, either alone or in combination with TNFα (Figure 1D). While deletion of Sucnr1 abolished the upregulation of these inflammatory mediators by TNFα alone, it failed to modify the response induced by co-treatment with TNFα and exogenous succinate, indicating that this co-stimulatory effect occurs independently of SUCNR1.

#### 2.1.3. Signaling Protein Expression

To characterize the signaling events underlying the functional changes described above, we analyzed the expression of key components of the NF-κB and MAPK pathways, which represent central pro-inflammatory routes associated with SUCNR1 activation [5].

The scrotal tissues of TNFα-treated WT animals showed increased levels of total and phosphorytlated NFκB, which left the ratio p-NFκB/NFκB unaffected. In contrast, tissues from *Sucnr1^−/−^* failed to augment total NFκB protein levels and presented a reduced p-NFκB/NFκB ratio in comparison with WT mice. Treatment with TNFα did not modify the total amount of ERK, but induced a non-significant increase in the p-ERK/ERK ratio in WT mice which was prevented in tissues from *Sucnr1^−/−^* animals. Finally, *Sucnr1^−/−^* mice presented lower levels of P38 while TNFα did not modulate the amount of P38 or p-P38 in any group (Figure 1E).

### 2.2. Influence of the Succinate-SUCNR1 Signaling Axis on Human Endothelial Cells In Vitro

To assess whether the findings observed in mice could be of relevance to humans and to distinguish the role of endothelial cells in those responses, we used human umbilical vein endothelial cells (HUVECs) treated with TNFα in the presence or absence of the SUCNR1 antagonist NF-56-EJ40. We then evaluated their interaction with human leukocytes (PBMCs) using a flow chamber assay and analyzed the expression of pro-inflammatory signaling proteins.

**Figure 2:**
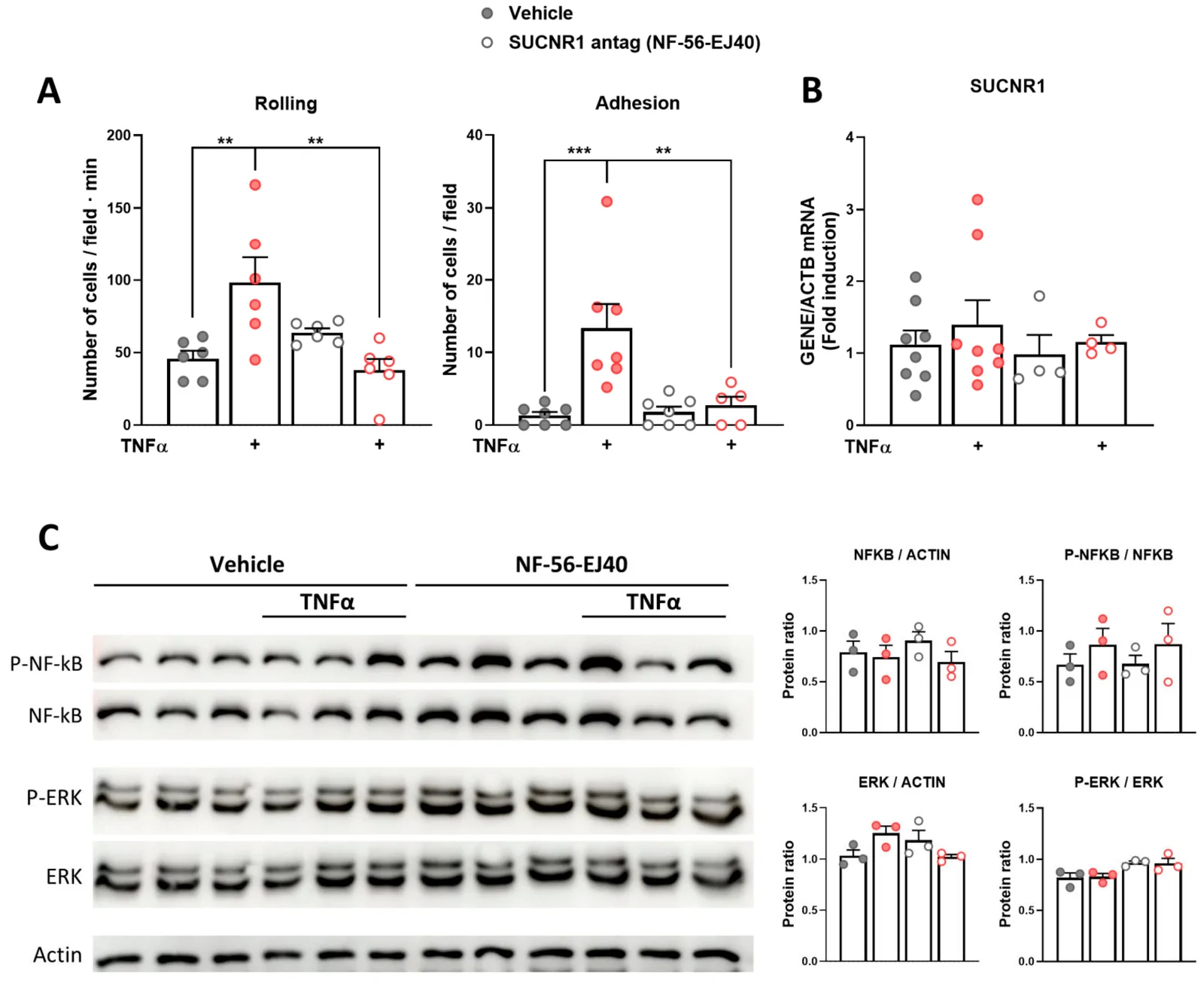
Effects of TNFα and a SUCNR1 antagonist on endothelial cells *in vitro*. HUVECs treated with TNFα (20 ng/mL) and/or the SUCNR1 antagonist NF-56-EJ40 (1 µm), or their corresponding vehicles (4 h), were placed in a parallel-plate flow chamber and the rolling and adhesion of superfused PBMC were analyzed under dynamic conditions ((**A**), n = 4–7, field of 0.54 mm^2^). Effects of these treatments on endothelial gene expression of SUCNR1 ((**B**), n = 4–7) and on signaling protein levels ((**C**), n = 3). Results are mean ± SEM, ** *p* < 0.01 and *** *p* < 0.001 (ANOVA followed by Tukey test).

As shown in Figure 2A, TNFα treatment of HUVECs significantly increased leukocyte rolling and adhesion. These interactions were markedly reduced by NF-56-EJ40 treatment (Videos S3 and S4). HUVECs treated with TNFα, in the presence or absence of the SUCNR1 antagonist, for 4 h showed normal levels of SUCNR1 mRNA. In parallel, the analysis of protein expression showed slight variations in the p-NFκB/NFκB ratio and the total amount of ERK which did not reach statistical significance in any case (Figure 2C).

**Figure 3:**
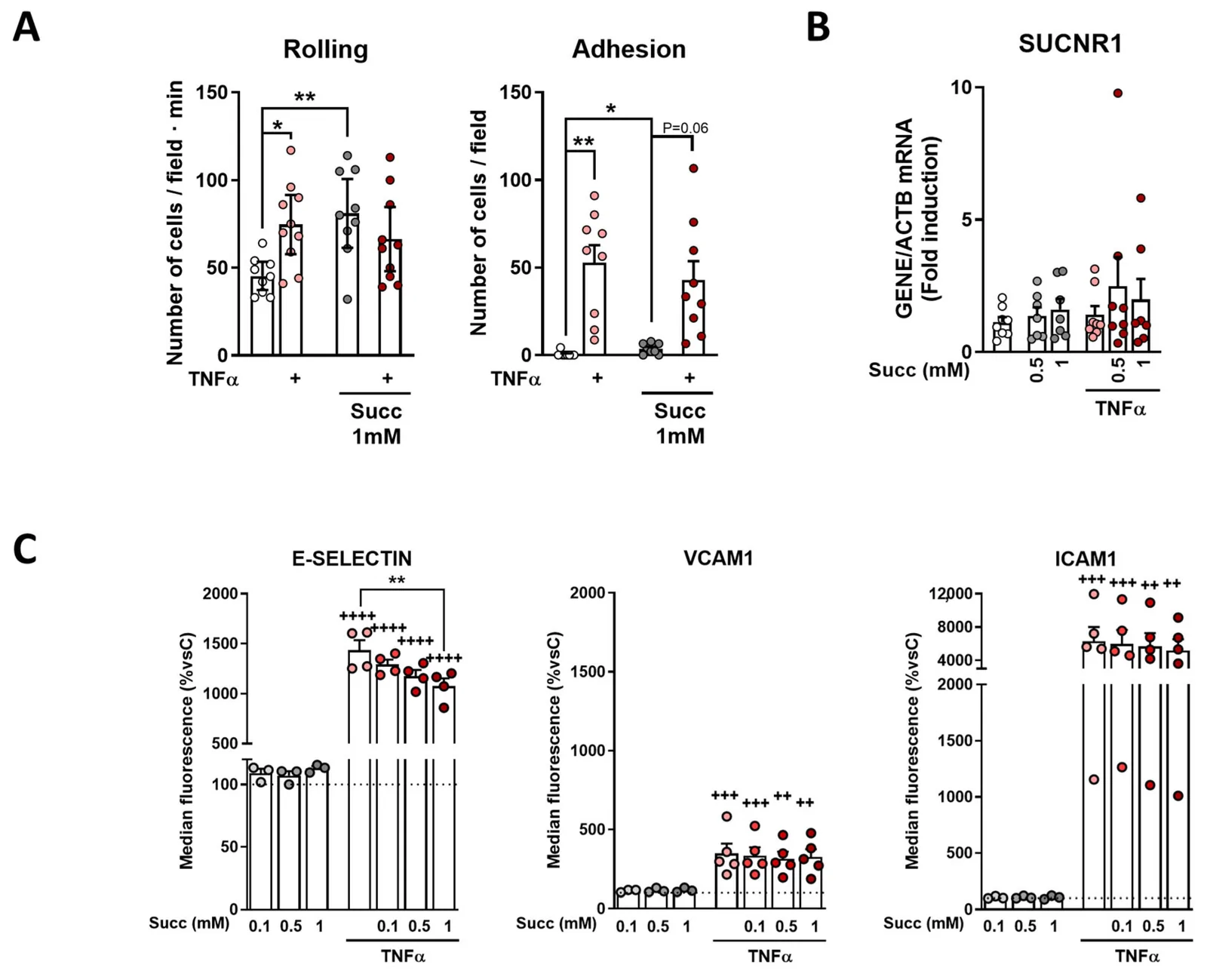
Effects of exogenous succinate on endothelial cells in vitro. HUVECs treated with TNFα (20 ng/mL) and/or succinate (0.1–1 mM), or their corresponding vehicles (4 h), were placed in a parallel-plate flow chamber and the rolling and adhesion of superfused PBMC were analyzed under dynamic conditions (n = 8–10, field of 0.54 mm^2^) (**A**). Effects of these treatments on endothelial gene expression of SUCNR1 (n = 7–8) (**B**) and on surface expression of adhesion molecules detected by flow cytometry (n = 3–5) (**C**). Results are mean ± SEM, * *p* < 0.05, and ** *p* < 0.01 vs. indicated group, ^++^ *p* < 0.01, ^+++^ *p* < 0.001 and ^++++^ *p* < 0.0001 vs. veh+veh group (ANOVA followed by Tukey test).

As in mice, we evaluated the effects of exogenous succinate on control and TNFα-treated endothelial cells. Succinate treatment of HUVECs increased PBMC rolling to an extent comparable to that induced by TNFα, while promoting PBMC adhesion to levels markedly lower than those observed in TNFα-treated cells. In contrast to this direct stimulatory effect, the addition of succinate to TNFα-treated cells appeared to attenuate the ability of TNFα to promote leukocyte–endothelial interactions (Figure 3A). Succinate did not modify SUCNR1 gene expression, either alone or in combination with TNFα (Figure 3B).

**Figure 4:**
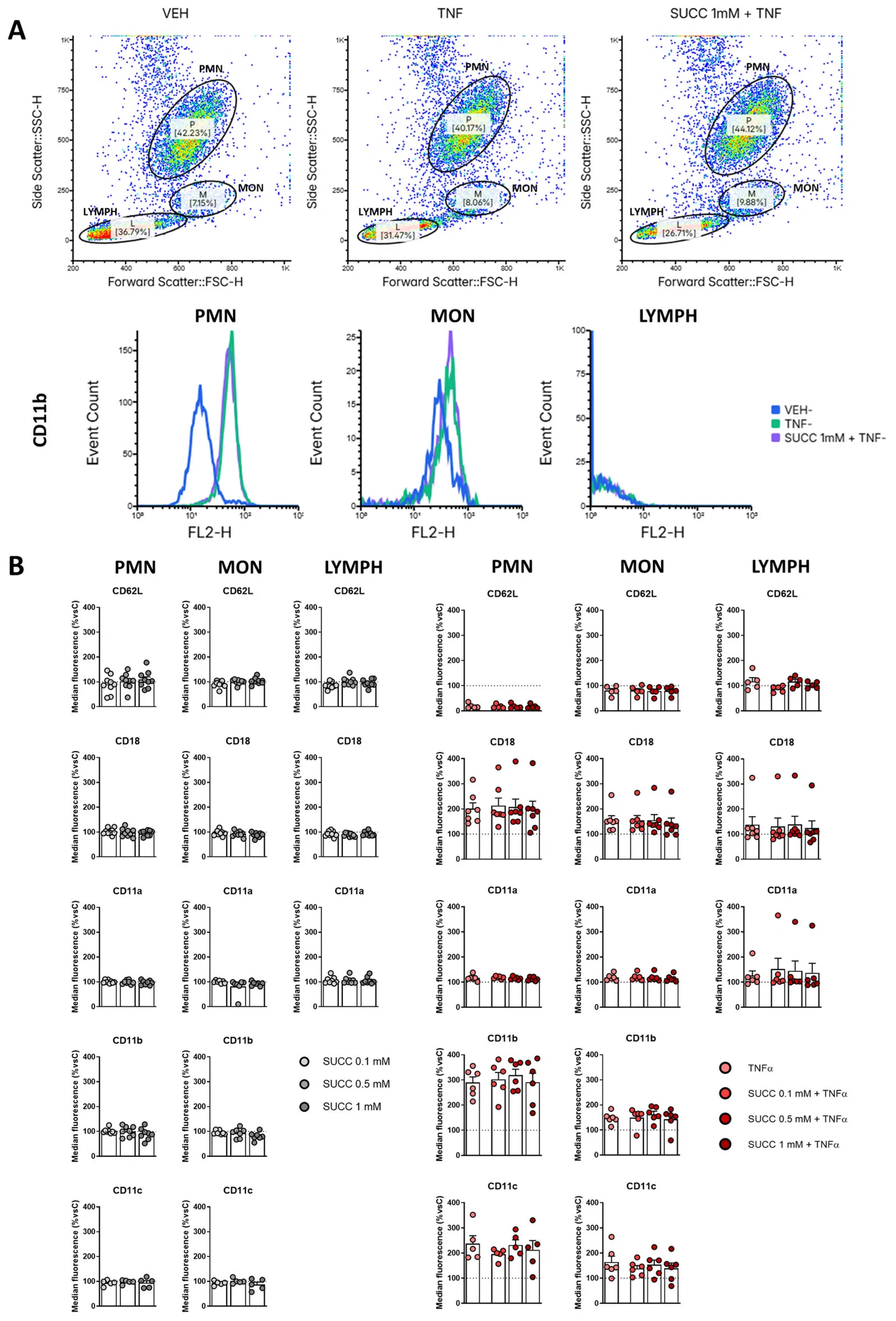
Effects of exogenous succinate on leukocytes from peripheral blood. The surface expression of adhesion molecules on polymorphonuclear leukocytes (PMN), monocytes (MON) or lymphocytes (LYMPH) from human blood samples treated with TNFα (20 ng/mL) and/or succinate (0.1–1 mM), or their corresponding vehicles (4 h), was analyzed by flow cytometry. (**A**) Representative images of the gating strategy and the resultant histograms. (**B**) Quantitative analysis of the fluorescent signal. Results are mean ± SEM, n = 7–9.

To explore the molecular mechanisms underlying these complex responses, we analyzed the effects of succinate, TNFα, and their combination on adhesion molecule expression in endothelial cells and leukocytes. Succinate-treated endothelial cells exhibited a modest, non-significant increase in surface E-selectin expression, whereas succinate addition to TNFα-treated cells significantly reduced E-selectin levels (Figure 3C). In contrast, none of the treatments altered VCAM-1 or ICAM-1 expression in endothelial cells (Figure 3C), nor the expression of adhesion molecules in human polymorphonuclear cells, monocytes, or lymphocytes (Figure 4) in basal or TNFα-stimulated conditions.

## 3. Discussion

Our results suggest that activation of the succinate receptor SUCNR1 may mediate the inflammatory effects of TNF-α by promoting leukocyte–endothelial interactions.

We observed that TNFα increases the protein expression of SUCNR1 and that TNFα-induced leukocyte rolling and adhesion on the venular endothelium are significantly reduced in *Sucnr1^−/−^* mice. These effects were associated with diminished inflammatory activity, as evidenced by lower expression of inflammatory cytokines and reduced NF-κB and ERK phosphorylation, along with an unanticipated reduction in *Sucnr1* gene expression. These findings point to a mediatory role for SUCNR1 signaling in TNFα-induced inflammation and suggest the activation of a negative feedback mechanism leading to reduced *Sucnr1* transcription.

TNFα has been reported to modulate the activity of enzymes directly involved in succinate metabolism [30,31,32,33,34], but we did not detect significant changes in global succinate levels in response to TNFα. Thus, the mediatory role of SUCNR1 on TNFα effects may be just due to the overexpression of this receptor which, according to the reported affinity and as suggested in previous studies [35], could get activated in response to basal succinate levels. Alternatively, the modulatory role of the succinate–SUCNR1 axis on TNFα activity may depend on alterations in the local distribution of succinate, a small, polar, and negatively charged metabolite whose transport across the plasma membrane relies on specific transporters that seem to be subject to tight regulation [9,36]. In this sense, we found that TNFα-induced the expression of *Slc26a6*, a membrane transporter that could promote the accumulation of succinate in the interstitial space, and thereby enhance SUCNR1 activation, by limiting the cellular uptake of succinate [16,28,29]. Exploratory correlation analyses revealed positive and significant associations between Slc26a6 upregulation and the expression of several pro-inflammatory cytokines and chemokines, providing preliminary support for this hypothesis. However, spatial analysis of succinate distribution by matrix-assisted laser desorption/ionization imaging mass spectrometry (MALDI-IMS) would be required to confirm it. In parallel, we observed that the local injection of a relatively high, but clinically relevant concentration of succinate [12], tended to intensify the inflammatory reaction induced by TNFα but limited its dependency on SUCNR1. In particular, we found that part of the leukocyte adhesion and the upregulation of *Tnfa* or *Il1b* expression induced by the combined treatment did not require the presence of this receptor. These responses could be driven by succinate through dual mechanisms, operating via mitochondrial ROS production through reverse electron transport at Complex I and by stabilizing HIF-1α through α-KGDDs inhibition in the cytosol [14,17]. Additionally, we cannot rule out the activation of other mechanisms in response to the expected change in local pH [37,38].

To analyze whether the observed response could be significant to human pathophysiology, we performed in vitro studies using human cells and observed that the leukocyte–endothelial interactions induced by TNFα were attenuated in the presence of a SUCNR1 antagonist. Thus, these results support the relevance of the succinate-SUCNR1 pathway in leukocyte infiltration during human inflammatory diseases characterized by elevated TNF-α levels, such as rheumatoid arthritis [11] or inflammatory bowel disease [12]. These results also suggest that the SUCNR1 proinflammatory activity observed in mice may involve a direct activation of the venular endothelium, although concomitant functional changes in other cell types are also likely and may contribute to the differences in signaling events observed between the *in vivo* and *in vitro* settings. For example, the response of resident macrophages to succinate and/or SUCNR1 activation [39,40,41], as well as the activation of polymorphonuclear neutrophils during extravasation [42,43], may explain why TNF induces a SUCNR1-dependent accumulation of total NF-κB in cremasteric tissues, whereas no corresponding increase is observed in HUVEC. NF-κB and ERK phosphorylation displayed a similar differential pattern, but these findings should be interpreted with caution. It is important to note that these experiments were exploratory, and phosphorylation was assessed 4 h post-TNF stimulation—matching the duration of functional assays—a time point at which transient phosphorylation events may have already subsided.

Finally, we tested the effects of exogenous succinate in endothelial cells and detected dual responses depending on the presence or absence of TNF-α, further reinforcing the context dependency of its biological activities described in previous studies [14,18,19,20]. Succinate increased leukocyte rolling and adhesion when administered alone, while the same treatment limited TNF-α-induced leukocyte–endothelial interactions and endothelial E-selectin expression. This effect may be related to the recently described ability of succinate to modify the endothelial cell surface [44], which could be particularly relevant for the dynamics of E-selectin. In this regard, and in comparison with ICAM-1 and VCAM-1, E-selectin displays a more transient surface presence following TNFα stimulation [45,46]. This is due to its more rapid proteolytic shedding and its high rate of constitutive endocytosis and lysosomal degradation, a process that depends on its location in clathrin-coated pits [47]. Moreover, its clustering in these pits and in lipid rafts facilitates its mediatory role on leukocyte rolling [47]. Succinate has been shown to induce lipid oxidation and membrane reorganization, promoting the activity of membrane-associated sheddases and leading to glycocalyx reduction [44]. These effects, described in the context of ischemia–reperfusion injury, where TNF-α plays a central role, may specifically modulate E-selectin function. In particular, membrane reorganization may impair the formation of clathrin-coated pits and lipid rafts that regulate E-selectin location, while increased metalloprotease activity may enhance E-selectin shedding. Both mechanisms could explain the reduced surface levels of E-selectin detected by flow cytometry and the limitation to the leukocyte–endothelial cell interactions observed in the flow chamber under succinate treatment. Alternatively, our results may reflect a shift from the Gq protein-activating conformation of SUCNR1 seen at low concentrations of succinate to a Gi protein-activating SUCNR1 conformation observed at higher extracellular succinate levels [48], which in our study may result from combining the exogenous administration with alterations in succinate distribution in response to TNFα. These questions will need to be addressed in future research.

In summary, our present results suggest that SUCNR1 stimulation may contribute to TNF-α-induced inflammation by acting on endothelial cells to promote leukocyte–endothelial interactions. This mechanism could act in concert with other known activities of succinate to nourish inflammation in multiple diseases; however, as is becoming increasingly clear, the pathogenetic role and the consequences of the blockade of the succinate-SUCNR1 axis may greatly vary depending on the context.

Limitations of this study: Two methodological aspects should be considered when interpreting our signaling data. First, the use of HUVECs (macrovascular) as a model for leukocyte–endothelium interaction may not fully capture the specialized responses of microvascular endothelial cells, which are the primary site of leukocyte recruitment in vivo. Second, protein phosphorylation was assessed 4 h post-TNF stimulation. Although this time point matches the peak expression of cell adhesion molecules, it may have missed earlier, transient phosphorylation peaks, thereby underestimating the magnitude of the initial signaling response. Future experiments evaluating phosphorylation within 30–60 min of treatment will be required to fully characterize the upstream signaling kinetics underlying the functional outcomes. Additionally, global SUCNR1 silencing in mice prevents determining the cell-specific contribution of endothelial SUCNR1 to TNFα-mediated activation in vivo.

## 4. Materials and Methods

### 4.1. Mice

*Sucrn1^−/−^* mice or wild-type (WT) mice (8–12 weeks old, 25–30 g weight, kindly provided by Dr Kenneth Mc Creath and Dr Ana Cervera [49]) were bred with a C57Bl/6 background. All animals were maintained under specific pathogen-free conditions in the animal facility of the University of Valencia and were co-housed to minimize potential effects of different microbiota.

### 4.2. Intravital Microscopy

We used intravital microscopy in the mouse cremaster muscle microcirculation, a method widely used to visualize blood cells interacting with the endothelium and within the vessels in vivo [27]. In brief, mice were anesthetized (xylacine hydrochloride, Sigma-Aldrich, St. Louis, MO, USA, 10 mg/kg and ketamine hydrochloride, Merial, Lyon, France, 100 mg/kg, intraperitoneally). The cremaster muscle was exteriorized onto an optical clear viewing pedestal for tissue transillumination and continuously superfused with bicarbonate-buffered saline (pH 7.4, 37 °C, 2 mL/min). It was visualized using an orthostatic microscope (Nikon Optiphot-1, SMZ1; Nikon, Badhoevedor, The Netherlands) onto which a video camera (Sony SSC-C350P; Sony, Koeln, Germany) was incorporated. Images (5 min period, ×1300) of single unbranched cremasteric venules [27], diameter 25–40 μm, measured with a video caliper (Microcirculation Research Institute, Texas A&M University, College Station, TX, USA), were captured digitally using the NIS Elements AR program (Version 5.x). Numbers of rolling and adherent leukocytes were determined offline during playback analysis of digital images by an observer who was unaware of the treatments administered. Rolling flux was assessed by counting the number of leukocytes passing a reference point in the vessel per minute. A leukocyte was considered to have adhered to the endothelium if it remained stationary for 30 s or more, and numbers were expressed per 100 μm of vessel [50].

Four hours before measurements, mice were treated with TNFα (Sigma-Aldrich, 500 ng) or its vehicle (100 µL, i.p.), and succinate (Sigma-Aldrich, 1 mM) or its vehicle (100 μL, intrascrotally). This route of administration was chosen to allow this solution to directly bathe the cremaster tissue with a clinically relevant succinate concentration [12].

### 4.3. Cell Culture

We employed passage one of human umbilical vein endothelial cells (HUVECs) harvested from human umbilical cords. Cells were treated (4 h, 37 °C) with TNFα (20 ng/mL) and/or succinate (0.1–1 mM), as appropriate. In some cases, cells were pre-treated with the SUCNR1 antagonist NF-56-EJ40 (MedChemExpress, Monmouth Junction, New Jersey, USA,−30 min, 1 µM, concentration selected from previous studies [19,51,52,53]). Peripheral blood mononuclear cells (PBMCs) were isolated from whole blood of healthy volunteers from the Hospital Clínico Universitario (Valencia) as previously described [54].

### 4.4. In Vitro Leukocyte/Endothelial Cell Interactions

The adhesion assay was performed under flow conditions using the parallel plate flow chamber model [54]. Coverslips [fibronectin (5 μg/mL) coated] containing confluent HUVEC monolayers were inserted in the chamber (37 °C) and a portion (5 × 25 mm) was exposed to the flow. The chamber was mounted on an inverted microscope (Nikon Eclipse TE 1000-S, ×40, Nikon, Badhoevedor, The Netherlands) fitted with a video camera (Sony Exware HAD, Sony, Koeln, Germany). PBMC were resuspended in buffer (Dulbecco’s PBS containing 0.1% human serum albumin) at 0.5 × 10^6^ cells/mL and were drawn across the monolayer (flow rate 0.36 mL/min, shear stress 0.7 dyne/cm^2^). Images of a single field (0.54 mm^2^) were recorded over a 5 min period and leukocyte parameters determined. Leukocyte rolling flux was calculated by counting the number of leukocytes rolling over the endothelial monolayer during a 1 min period (expressed as cells/min). Leukocyte adhesion was determined by counting the number of leukocytes that maintained stable contact with the monolayer for 30 s in a total of five high-power fields (5 × 0.54 mm^2^).

### 4.5. RNA Isolation and Real-Time Quantitative PCR (RT-qPCR)

Cremasteric tissues from WT or *Sucnr1*^−/−^ mice were homogenated (ULTRA-TURRAX^®^, IKA, Staufen, Germany). Total RNA from mouse tissue and cells were obtained using the extraction kit (Illustra RNAspin Mini, GE HealthCare Life Science, Barcelona, Spain) according to the manufacturer’s instructions. For reverse transcription, cDNA was obtained with the Prime Script RT reagent Kit (Takara Biotechnology, Dalian, China). Quantitative PCR (qPCR) was performed with the Prime Script Reagent Kit Perfect Real Time (Takara Biotechnology, Saint-Germain-en-Laye, France) in a thermo cycler Light Cycler (Roche Diagnostics, Mannheim, Germany). Results were expressed as fold increase calculated as follows: change in expression (fold) = 2^−Δ(ΔCT)^ where ΔCT = CT (target) − CT (housekeeping) and Δ(ΔCT) = ΔCT (treated) − ΔCT (control). In all cases, the housekeeping gene used was β-actin and the experiments were performed in duplicate. Specific primers were designed according to the sequences present in Table A1.

### 4.6. Protein Extraction and Western Blot Analysis

Proteins from tissues and HUVECs were obtained and the expression of several proteins were analyzed by Western blot as previously described [55]. Equal amounts of protein were loaded onto SDS/PAGE gels. Membranes were incubated with specific primary antibodies (Table A2) and with a peroxidase-conjugated anti-mouse IgG (Thermo Scientific, Rockford, IL, USA, 1:2000) or anti-rabbit IgG (Thermo Scientific, 1:5000). The signal was detected using supersignal west pico chemiluminescent substrate (Thermo Scientific) and an Amersham™ ImageQuant™ 800 biomolecular imager (GE Healthcare, Chicago, IL, USA). Phosphorylated forms of the analyzed proteins were initially detected, after which membranes were stripped and reprobed for the corresponding total protein levels, allowing assessment of both phosphorylated and total protein expression on the same membrane. The Image Gauge version 4.0 software (Fujifilm) was used to quantify the protein expression by means of densitometry. Total protein data were normalized to Actin.

### 4.7. Succinate Quantification

The Succinate Assay Kit (Abcam, Cambridge, UK) was used according to the manufacturer’s instructions to measure succinate in murine cremasteric tissue. Briefly, tissues were homogenized and centrifuged, and the resultant supernatants were filtered with 10 kDa spin columns (ab93349, Abcam, Cambridge, UK). The Reaction Mix from the kit was used to incubate samples in 96-well plates at 37 °C during 30 min. Finally, the absorbance at 450 nm was measured with the microplate reader SpectraMax Plus 384 (Molecular Devices, San Jose, CA, USA) and the succinate concentration was calculated using the standard curve.

### 4.8. Flow Cytometry

HUVEC monolayers incubated in different conditions were detached from the culture dish with trypsin and, after washing, were incubated (20 min, 4 °C, in darkness) with saturating amounts of fluorescein or phycoerythrin conjugated antibodies against endothelial adhesion molecules (CD62E—E-selectin; VCAM1—Vascular cell adhesion protein; ICAM1—Intercellular adhesion molecule 1, all from BD Biosciences, San Jose, CA, USA).

Leukocyte adhesion molecules (CD11a—LFA-1; CD11b—Mac-1; CD11c—gp150.95; CD18—β2-integrins; CD62L—L-selectin) were analyzed in citrated blood samples. These samples were incubated (20 min, 4 °C, in darkness) with saturating amounts of the corresponding fluorescein or phycoerythrin conjugated antibodies (all from BD Biosciences). Subsequently, the samples were lysed and fixed with the BD FACS Lysing Solution (BD Biosciences, San Jose, CA, USA).

The resultant cell suspensions were analyzed in a flow cytometer (FACSCalibur, BD Biosciences). Neutrophils, monocytes, and lymphocytes were identified by their specific features of size (forward-angle light scatter) and granularity (side-angle light scatter). The median of the specific fluorescence intensity was considered a marker of the expression of the respective epitope. Experiments were performed in duplicate, and ten thousand cells were analyzed in each sample.

### 4.9. Statistical Analysis

Data are presented as mean ± SEM, and normality tests were used to check their distribution. One-way ANOVA followed by Tukey’s test or Kruskal–Wallis followed by Dunn’s test were used, as appropriate, to compare three or more experimental groups. T-test analysis or Mann–Whitney’s test were used, as appropriate, to analyze the experiments with only two groups. Spearman’s rank correlation was used to assess associations between variables and P values were corrected for multiple comparisons using the Benjamini–Hochberg FDR method. Adjusted *p*-values under 0.05 were considered significant.

## Data Availability

The original contributions presented in this study are included in the article/Supplementary Material. Further inquiries can be directed to the corresponding author.

## Supplementary Materials

The following supporting information can be downloaded at: https://www.mdpi.com/article/10.3390/ijms27177930/s1.

## Abbreviations

The following abbreviations are used in this manuscript:

α-KGDD	Alpha-ketoglutarate-dependent dioxygenase
SUCNR1	Succinate receptor 1
TNFα	Tumor necrosis factor alpha
HUVECs	Human umbilical vein endothelial cells
PBMCs	Peripheral blood mononuclear cells
CD11a—LFA-1	Integrin alpha-L
CD11b—Mac-1	Integrin alpha-M
CD11c—gp150.95	Integrin alpha-X
CD18	Integrin beta-2
CD62E	E-selectin
CD62L	L-selectin
ERK	Mitogen-activated protein kinase 3
ICAM1	Intercellular adhesion molecule 1
α-KGDDs	α-Ketoglutarate-dependent dioxygenases
LYMPH	Lymphocytes
MON	Monocytes
NFκB	Nuclear factor NF-kappa-B p65 subunit
p38	Mitogen-activated protein kinase 11
PMN	Polymorphonuclear leukocytes
SDH	Succinate dehydrogenase
VCAM1	Vascular cell adhesion protein 1
WT	Wild-type

## Appendix A

**Table A1:** Sequences for the primers used in Real-Time Quantitative PCR (RT-qPCR) studies.

	Target	SEQUENCE	
		(s)	(as)
Murine	*Cxcl1*	CGAAGTCATAGCCACACTCAAG	TCTCCGTTACTTGGGGACAC
	*Cxcl5*	TGCCCCTTCCTCAGTCATAG	AGACCTCCTTCTGGTTTTTCAG
	*Il1b*	TGCCACCTTTTGACAGTGATG	ATGTGCTGCTGCGAGATTTG
	*Slc13a3*	GAGGAACACCGCAGAAACATC	CCACCAGTAGGAACAACAGCA
	*Slc26a6* (*isoform 1*)	GGTGTAGATGTTGACCGCCT	ATCCTGAAGCTCCTCCCCTT
	*Slc26a6* (*isoform 2*)	GTCTTCTCCTTGCTGCTCGT	TCAACATCTACACCGCACTTCT
	*Sucnr1*	TCACTGTGGTGTTTGGCTACCT	CCCTTATCATTGGCATAACTCTTTATC
	*Tnfa*	ATCGGTCCCCAAAGGGATG	GGTGGTTTGTGAGTGTGAGGG
Human	SUCNR1	GGAGACCCCAACTACAACCTC	AGCAACCTGCCTATTCCTCTG

**Table A2:** Antibodies used in Western blot analysis.

Target	Antibody Reference	Dilution
ACTIN	A5060, Sigma-Aldrich, St. Louis, MO, USA	1:5000
ERK	4695, Cell Signaling, Danvers, MA, USA	1:1000
NF-κB p65	8242, Cell Signaling	1:1000
Phospho-ERK	9101, Cell Signaling	1:1000
Phospho-NF-κB p65	3033S, Cell Signaling	1:1000
p38	506123, Sigma-Aldrich	1:1000
Phospho-p38	28B10, Cell Signaling	1:1000
SUCNR1	NBP1-00861, Novus Biologicals, Centennial, CO, USA	1:1000
